# Electrical Stimulation Decreases Dental Pulp Stem Cell Osteo-/Odontogenic Differentiation

**DOI:** 10.1089/biores.2020.0002

**Published:** 2020-05-27

**Authors:** Karla Mychellyne Costa Oliveira, Liudmila Leppik, Khyati Keswani, Sreeraj Rajeev, Mit B. Bhavsar, Dirk Henrich, John H. Barker

**Affiliations:** ^1^ Frankfurt Initiative for Regenerative Medicine, J.W. Goethe-University, Frankfurt/Main, Germany.; ^2^ Department of Trauma-, Hand- and Reconstructive Surgery, J.W. Goethe-University, Frankfurt/Main, Germany.

**Keywords:** cell differentiation, dental pulp stem cells, electrical stimulation, *in vitro*, osteogenic differentiation

## Abstract

Dental pulp stem cells (DPSCs) have great potential for use in tissue engineering (TE)-based dental treatments. Electrical stimulation (EStim) has been shown to influence cellular functions that could play an important role in the success of TE treatments. Despite many recent studies focused on DPSCs, few have investigated the effect EStim has on these cells. The aim of this research was to investigate the effects of direct current (DC) EStim on osteo-/odontogenic differentiation of DPSCs. To do so cells were isolated from male Sprague Dawley rats (7–8 weeks old), and phenotype characterization and multilineage differentiation analysis were conducted to verify their “stemness.” Different voltages of DC EStim were administrated 1 h/day for 7 days, and the effect of EStim on DPSC osteo-/odontogenic differentiation was assessed by measuring calcium and collagen deposition, alkaline phosphatase (ALP) activity, and expression of osteo- and odontogenic marker genes at days 7 and 14 of culture. We found that while 10 and 50 mV/mm of EStim had no effect on cell number or metabolic activity, 100 mV/mm caused a significant reduction in cell number, and 150 mV/mm resulted in cell death. Despite increased gene expression of osteo-/odontogenic gene markers, *Osteocalcin*, *RunX2*, *BSP*, and *DMP1*, at day 7 in EStim treated cells, 50 mV/mm of EStim decreased collagen deposition and ALP activity at both time points, and calcium deposition was found to be lower at day 14. In conclusion, under the conditions tested, EStim appears to impair DPSC osteo-/odontogenic differentiation. Additional studies are needed to further characterize and understand the mechanisms involved in DPSC response to EStim, with an eye toward its potential use in TE-based dental treatments.

## Introduction

Alveolar bone, one of the major structures of the periodontium, provides important structural support for teeth and dental implants. As such it is constantly subjected to external mechanical stresses that cause its continuous remodeling and resorption.^1^ Since spontaneous regeneration does not occur in alveolar bone, damage caused by trauma or periodontitis generally requires surgical interventions that may result in tooth loss and the need for bone grafting, accompanied by its associated risks (reviewed in Ref.^2^). Therefore, bone regeneration in cases of reduced alveolar bone continues to be a challenge for maxillofacial surgeons and patients requiring prosthodontics and implant treatments.

An increasing number of studies support the use of tissue engineering (TE) as an excellent alternative treatment option for bone reconstruction in the fields of dentistry and medicine. The success of these new TE therapeutic strategies depends on the positive interplay between stem/progenitor cells, scaffolds, and growth factors (reviewed in Refs.^3,4^). While mesenchymal stem cells (MSCs) from multiple origins have been investigated, bone marrow-derived MSCs (BM-MSCs) continue to be the most commonly used cell source in TE applications.^4,5^ Commonly cited drawbacks associated with the use of BM-MSCs in TE applications include donor site morbidity, infection, and the fact that proliferation and differentiation capacities depend on donor characteristics (reviewed in Ref.^6^). Since dental pulp stem cells (DPSCs) were first identified and described, in 2000,^7^ their potential for overcoming the limitations associated with BM-MSCs has been recognized (reviewed in Ref.^8^). A few of their important qualities that have supported this claim include their easy extraction from pulp tissue, no morbidity or ethical concerns associated with their harvest (reviewed in Ref.^9^), and great plasticity and regenerative capacity.^10^ DPSCs are similar to BM-MSCs in their ability to repair musculoskeletal tissues (reviewed in Ref.^8^), good compatibility with biomaterials,^11^ and their demonstrated ability to promote bone formation in mandibular defects.^12^ These characteristics make DPSCs a good candidate for use in guided bone regeneration treatments.

Electrical stimulation (EStim) has been used successfully in the field of orthopedics to accelerate healing in recalcitrant nonhealing bone defects. Recent reports in the literature have shown that EStim influences stem cell migration, proliferation, osteogenic differentiation, and attachment to scaffolds, all cell behaviors that are key to the success of bone TE treatments.^13–18^ In *in vitro* model systems, others and we have demonstrated improved osteogenic differentiation of MSCs after EStim treatment.^13,15,18,19^ Specifically, we exposed BM-MSCs, and separately adipose-derived MSCs (AT-MSCs), to direct current (DC) EStim and observed increased calcium deposition and expression of osteogenic genes in treated cells.^13,15^ In separate experiments we reproduced this beneficial effect in a rat femur large defect model, where we demonstrated that EStim treatment improved bone healing and regeneration.^14,20^ EStim has also been shown to increase bone formation at the bone–implant interface in dental implants.^21,22^

Few studies exist that test the effect of EStim on DPSCs or the concept of combining DPSCs and EStim for TE applications.^23^ Combined with TE approaches for alveolar bone regeneration, EStim could potentially shorten waiting periods in guided bone regeneration treatments, implant surgery, and initiation of prosthetic procedures. In this study, we isolated and characterized rat DPSCs and analyzed the influence EStim has on their osteo-/odontogenic differentiation, with an eye toward using EStim to optimize DPSC-based TE dental treatments.

## Materials and Methods

All animal experiments were performed in accordance with guidelines established by our Animal Care And Oversight Committee at the Johann Wolfgang Goethe-University in Frankfurt am Main, according to German law.

### Cell isolation and culture

DPSCs were isolated from lower incisors of Sprague Dawley rats as previously described^10^ (see Supplementary Data for details).

#### Characterization of isolated DPSCs.

##### Phenotype characterization

Isolated DPSCs were assessed for stem cell marker expression using flow cytometry analysis. Surface protein CD90, typically expressed in rat DPSCs, was used as a positive marker; hematopoietic stem cell markers CD45 and CD34 were used as negative controls (Supplementary Data).

##### Multilineage differentiation

DPSC osteo-/odontogenic and chondrogenic differentiation were induced chemically for 21 days as described in Supplementary Data.

#### EStim of DPSCs

Cells were exposed to DC EStim in a purpose-built EStim cell culture chamber, as described elsewhere.^24,25^ DPSCs (1.25 × 10^4^ cells/cm^2^) were seeded in six-well plates in osteo-/odontogenic medium and exposed to different voltages (10, 50, 100, and 150 mV/mm) of EStim 1 h/day during 7 days. Non-EStim treated cells were used as controls. All experiments were performed in triplicates.

##### EStim Cytotoxicity

Before conducting the experiments, we verified that the different EStim voltages tested were not cytotoxic to DPSCs, by measuring cell numbers and metabolic activity. Measurements were performed in cells treated with different EStim voltages (10, 50, 100, and 150 mV/mm) and in nontreated cells (control) at day 7 of culture, in triplicates. In addition, the effect that these different EStim voltages had on cell morphology was assessed using an Olympus CKX41 light microscope (Tokyo, Japan).

*Cell number* measurements were facilitated using PicoGreen assay (Quant-iT™ *PicoGreen*^®^; Thermo Fisher) according to the manufacturer's protocol (see Supplementary Data).

*Cell metabolic activity* was evaluated by alamarBlue Assay (alamarBlue^®^ Cell Proliferation Assay Kit; Bio-Rad, Germany) following the manufacturer's instructions (see Supplementary Data). The percentage of reduction was calculated using the equation provided by the manufacturer, and values were normalized by the number of cells in each well. The mean value for three wells was calculated for each group and used for statistical analysis.

#### Assessment of DPSC osteo-/odontogenic differentiation

The effect of EStim on DPSC osteo-/odontogenic differentiation was evaluated at days 7 and 14 of culture by means of collagen and calcium deposit staining, alkaline phosphatase (ALP) activity, and osteo-/odontogenic marker gene expression analysis (Supplementary Table S1).

*Collagen formation* was examined in EStim treated and control cells at days 0 (1 day after seeding), 7, and 14 using Picrosirius Red staining as in our previous study,^13^ briefly described in Supplementary Data.

*Calcium deposits* were evaluated in EStim treated and nontreated (control) cells at day 7 and 14 using Alizarin Red staining, as described in Supplementary Data. Stained cells were imaged with a light microscopy (Olympus CKX41) and cellSens Entry 1.9 software.

*ALP activity* was measured in cell lysates prepared in the same way as for cell number quantification. SensoLyte pNPP Alkaline Phosphatase Detection Kits (Anaspec, Inc., California, USA) were used according to the manufacturer's instructions (Supplementary Data).

*Osteo-/odontogenic marker gene expression analysis* was performed using RT-qPCR. Total RNA was isolated from cells, and cDNA synthesis was conducted. Quantitative real-time polymerase chain reaction was performed with a CFX96 Touch Real-Time PCR Detection System (Bio-Rad) (Supplementary Data).

### Statistical analysis

JMP13 software (Statistical Discovery; SAS Institute, Inc.) was used to determine statistical significances. For experiments comparing different voltages, statistical significance was determined by one-way ANOVA, followed by Tukey *post hoc* test. Student's *t*-test was used for comparisons between treated and nontreated groups at different time points. All experiments were performed in triplicates, and differences were considered to be significant at 95% (*p < 0.05).

## Results

### DPSC characterization

One day after isolation seeded DPSCs became elongated in shape, organized into colonies, and attached to the bottom of the plates and, after 6 days, reached confluence in the culture plates.

#### Phenotype characterization (flow cytometry)

Immunophenotype characterization revealed that the isolated DPSCs tested positive for surface marker CD90 (96.12%), and hematopoietic cell markers CD45 and CD34 were found on less than 17% of cells (Supplementary Fig. S1A). 7AAD stain showed that 98.2% of cells analyzed were viable (Supplementary Fig. S1A).

#### Multilineage differentiation

Isolated DPSCs demonstrated successful differentiation capacity into osteo-/odontogenic and chondrogenic cell lineages. DPSC osteo-/odontogenic differentiation was confirmed at day 21 of osteogenic culture by Alizarin Red staining of calcium deposits. Strong mineralization of extracellular matrix was observed in DPSCs cultured in osteo-/odontogenic medium compared to those cultured in normal medium (Supplementary Fig. S1Bi). Chondrogenic differentiation of DPSC was observed in cells cultured in pellet culture under chondrogenic conditions. The deposition of sulfated glycosaminoglycans was detected using dimethylmethylene blue staining in DPSC cell pellets at day 21 of culture (Supplementary Fig. S1Bii).

### EStim cytotoxicity

To identify the optimal EStim voltage, DPSCs were exposed to different EStim voltages (10, 50, 100, and 150 mV/mm) over a period of 7 days, and cell morphology, metabolic activity, and cell number were evaluated.

**FIG. 1.**
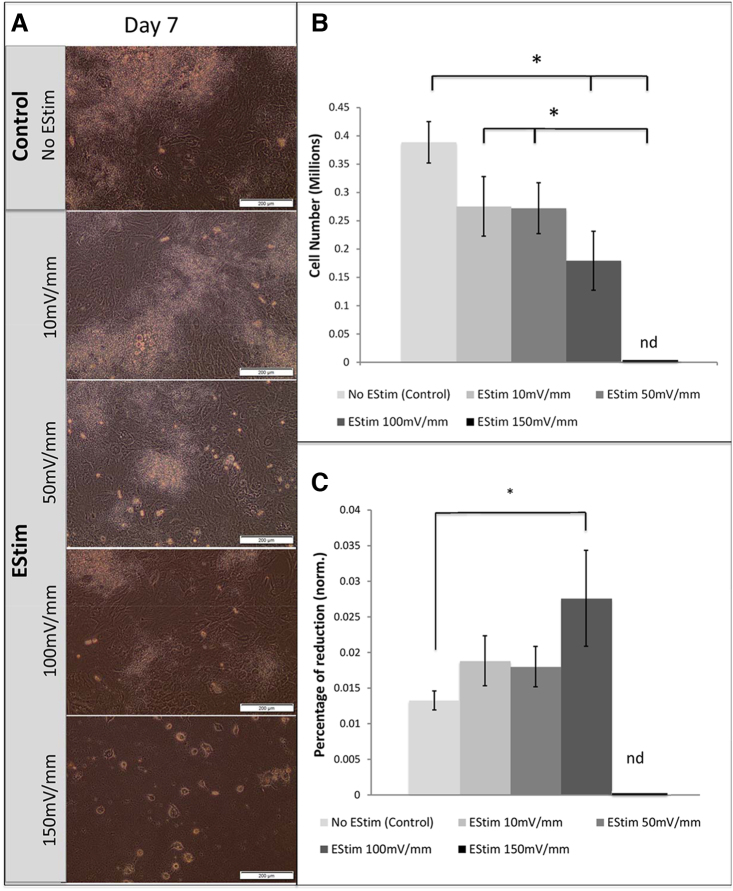
Cell morphology, number, and metabolic activity following treatment with different EStim voltages. **(A)** Morphology of cells nontreated and treated with different EStim voltages at 7 days of culture (10 × magnification; Scale bar = 200 μm). **(B)** Cell number measured in different treatment groups at day 7 of culture. The maximum number of cells was seen in the control group. 150 mV/mm of EStim significantly reduced cell number to values below detection levels (*p < 0.05). **(C)** Metabolic activity after 7 days of EStim treatment with different voltages showed significant difference between the control and 100 mV/mm EStim groups. For 150 mV/mm, measurements were not possible due to cell death (*p < 0.05). EStim, electrical stimulation; nd, not detected.

*Morphology analysis* of cells treated with different EStim voltages revealed that 150 mV/mm negatively impacted the cells, as treated cells were less confluent than cells in the other treatment groups and mostly detached from the plate surface (Fig. 1A). In the groups receiving lower voltages, deposits over differentiating cells were visible; however, the number of these deposits decreased with increased EStim voltage (Fig. 1A). The maximum number of cell deposits was observed in nontreated control cells, and the minimum was in those treated with 100 mV/mm EStim (Fig. 1A).

*Cell numbers* in groups receiving 10 and 50 mV/mm of EStim did not differ from the nontreated group at day 7. On the same day 100 mV/mm caused significant decrease in cell number, and 150 mV/mm led to cell death (Fig. 1B).

*Metabolic activity* measurements in cells treated with different voltages for 7 days revealed that 10 and 50 mV/mm did not significantly affect cell activity compared to nontreated controls, whereas cells exposed to 100 mV/mm experienced an increase in cellular activity compared to controls (Fig. 1C). In cells exposed to 150 mV/mm, cell activity was not detectable since this voltage led to cell death.

Based on these results, 50 mV/mm of EStim was taken to be optimal and was used in the subsequent experiments.

### Assessment of osteo-/odontogenic differentiation

**FIG. 2.**
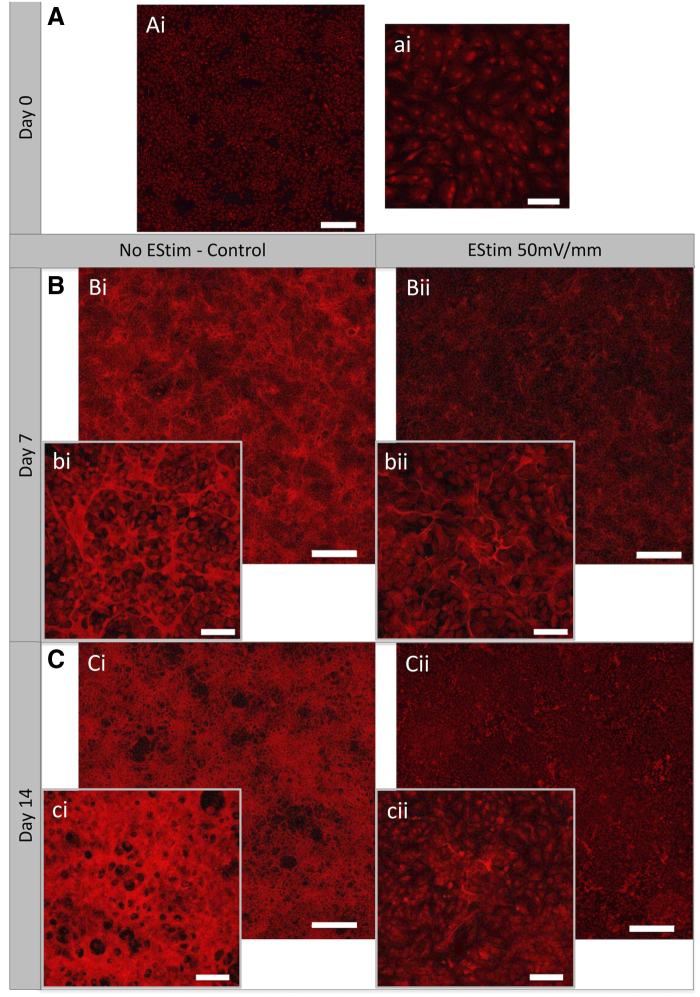
Collagen fibrils and ECM networking in EStim treated and nontreated (control) cells at different time points. **(Ai, ai)** Representative images showing absence of collagen fibrils and ECM network at day 0 (scale bar = 500 and 100 μm, respectively). **(B)** Representative images from both groups at day 7: **(Bi)** nontreated cells show collagen fibers forming a network on the surface of cells (scale bar = 500μm); **(bi)** higher magnification (10 × ) image of nontreated cells shows densely packed and connected collagen fibrils (scale bar = 100 μm); **(Bii)** EStim treated cells exhibit low amounts of collagen fibers and poorly formed collagen network (scale bar = 500 μm); **(bii)** higher magnification (10 × ) image of EStim treated cells shows thin collagen fibrils formed over the cells (scale bar = 100 μm). **(C)** Collagen staining at day 14: **(Ci)** strong extracellular collagen network is visible over nontreated cells (scale bar = 500 μm); **(ci)** higher magnification image of nontreated cells shows strong collagen network forming into a lamellar configuration (scale bar = 100 μm); **(Cii)** cells treated with EStim show absence of collagen network formation (scale bar = 500 μm); **(cii)** higher magnification image shows low amount of collagen fibrils over cells (scale bar = 100 μm). ECM, extracellular matrix.

*Collagen deposition* was visualized with Sirius red staining and measured at high magnification by assessing variation in collagen fiber thickness and network distribution in EStim treated and nontreated cells at days 7 and 14 of osteo-/odontogenic culture. At day 0 intracellular collagen was visible; however, extracellular collagen fibers and network organization were not detected (Fig. 2A). At day 7, in nontreated cells, collagen fibrils appeared densely packed and well organized, and a collagen network could be seen in some areas between the cells (Fig. 2B). In EStim-treated cells, collagen fibrils appeared thinner, and fiber networks were not as evident as in controls (Fig. 2Bbi, bii). In controls, after 14 days of culture, collagen network appeared more compact covering most of the cells, while in the EStim treated cells only in some areas was collagen deposition visible (Fig. 2C).

**FIG. 3.**
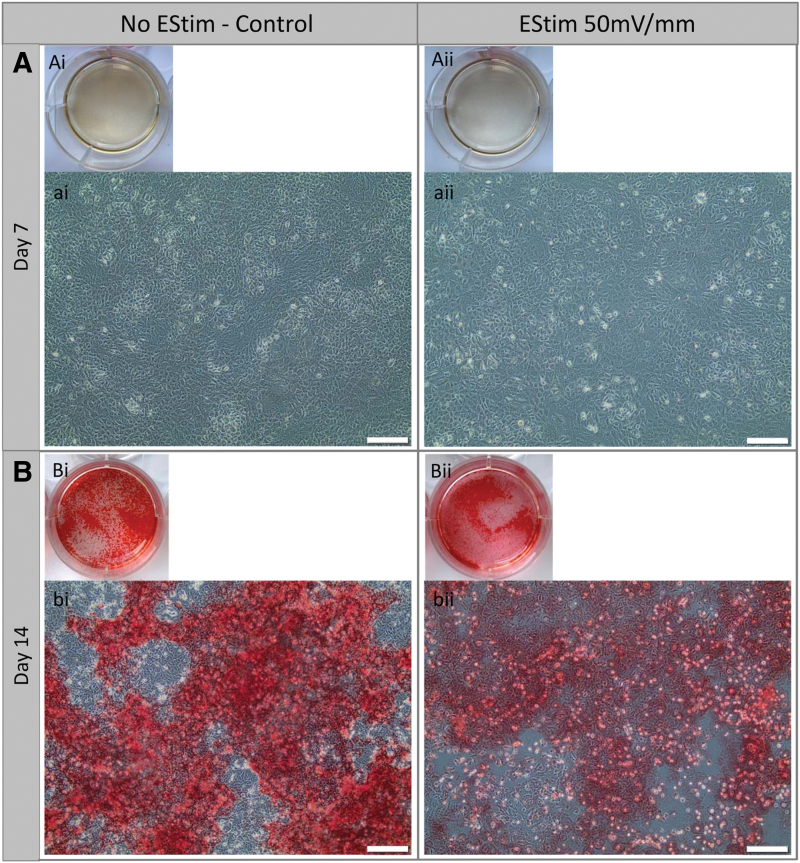
Alizarin red-stained calcium deposits of EStim treated and nontreated (control) cells at days 7 **(A)** and 14 **(B)** of osteogenic culture. **(Ai, Aii)** Overview of cell culture wells showing absence of calcium deposition (no red color) at day 7 in nontreated and EStim treated cells, respectively. **(ai, aii)** High magnification representative images of EStim treated and nontreated cells showing absence of calcium deposits (scale bar = 200 μm). **(Bi, Bii)** Overview of cell culture wells showing the presence of calcium deposits at day 14 in EStim treated and nontreated cells. **(bi, bii)** High magnification representative images showing robust calcium deposits formed in nontreated cells **(bi)** and moderate calcium deposits in EStim treated cells **(bii)** (scale bar = 200 μm).

*Calcium deposition* at 7 and 14 days of osteo-/odontogenic culture was investigated in cells treated daily with EStim or nontreated (control) cells. In both groups calcium deposition (stained Red) was detected only at day 14 of culture (Fig. 3) and appeared to differ between groups. While nontreated cells showed strong and homogeneous calcium deposit distribution, cells treated with EStim showed lower amounts of calcium deposition, concentrated in select focused areas (Fig. 3).

**FIG. 4.**
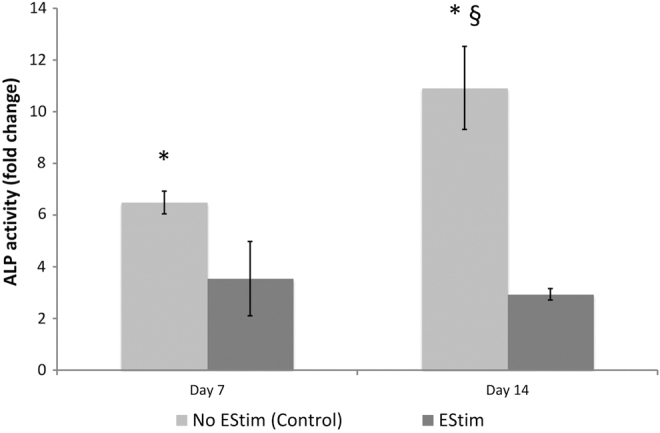
ALP activity in EStim treated and nontreated cells at days 7 and 14 of culture. ALP activity was decreased by EStim treatment at both the 7- and 14-day time points. Comparisons between time points revealed that ALP activity was increased in nontreated cells and remained stable in EStim treated cells at the later time point. Asterisk (*) indicates significant differences between groups within the same time point. Section sign (§) indicates significant differences between time points within the same group (*p* < 0.05). ALP, alkaline phosphatase.

*ALP activity* was significantly decreased in EStim treated cells, compared to nontreated cells, at both 7 and 14 days. In the latter, ALP activity significantly increased over time, whereas in the EStim group values did not differ between 7 and 14 days of culture (Fig. 4).

**FIG. 5.**
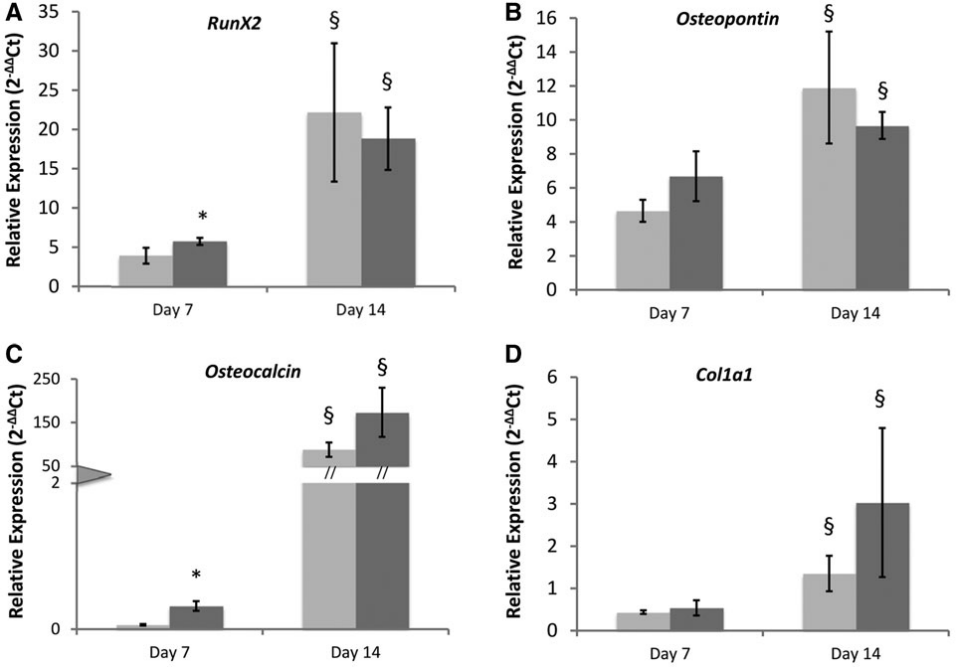

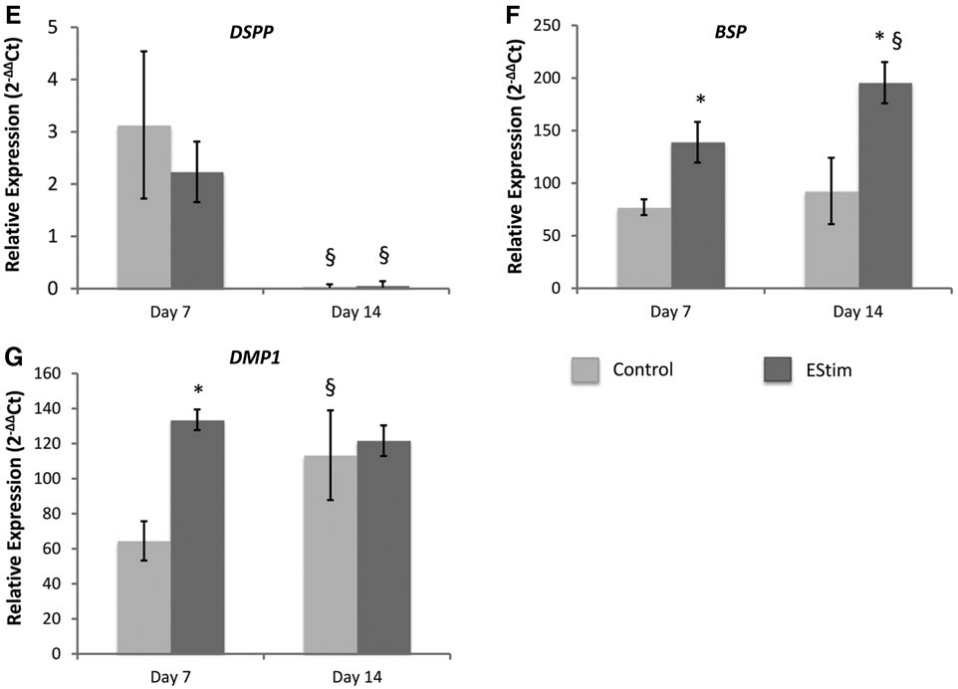
Osteo/odontogenic marker gene expression in EStim treated and nontreated DPSCs at days 7 and 14 of culture. **(A)** Expression of *RunX2*, **(B)** *Osteopontin*, **(C)** *Osteocalcin*, **(D)** *Col1a1*, **(E)** *DSPP*, **(F)** *BSP*, and **(G)** *DMP1* genes measured by mean of RT-qPCR at 7 and 14 days of culture. Asterisk (*) indicates significant differences between groups within the same time point. Section sign (§) indicates significant differences between time points within the same group (*p* < 0.05). DPSC, dental pulp stem cell; RT-qPCR, reverse transcriptase quantitative polymerase chain reaction.

*Osteo-/odontogenic marker gene expression:* There was no difference in the expression levels of *Osteopontin* and *Col1a1* genes, in cells treated with 50 mV/mm compared to nontreated controls, at days 7 and 14 of culture. Expression of *RunX2* and *Osteocalcin* was significantly increased at day 7 in EStim treated cells. *RunX2, Osteopontin, Osteocalcin,* and *Col1a1* expression were found to be upregulated from day 7 to 14 in both EStim and control groups. At the same time, expression of specific odontogenic gene markers was shown to be influenced by EStim. EStim significantly increased the expression of *BSP* gene at both time points and expression of *DMP1* at day 7 of culture. Expression of *DSPP* gene was not affected by EStim; however, it decreased over time in both treated and nontreated cells (Fig. 5).

## Discussion

*In vitro* studies have demonstrated EStim's ability to enhance specific cell activities that could be used to improve the effectiveness of TE treatments (reviewed in Ref.^26^). While in dental medicine, EStim has not traditionally been used to stimulate bone regeneration, an increasing number of studies have shown that electrically charged scaffolds and conductive dental implants can improve osteointegration and bone formation.^19,21,22^ Recently, Cheng et al. demonstrated that electrically conductive films increased osteogenic differentiation of human DPSCs.^23^ Despite these recent findings, exactly how EStim influences dental tissue-derived stem cells is poorly understood. In this study, we investigated the effects EStim has on osteo-/odontogenic potential of DPSCs, with an eye toward its potential use in combination with TE approaches for improving outcomes in dental treatments.

DPSCs were isolated from rat lower incisors, and their “stemness” was verified by surface marker expression and multilineage differentiation analysis. As sensitivity of DPSCs to EStim was not described previously, it was important to first perform a dose titration to determine which voltage(s) were effective and/or cytotoxic. Considering the voltage range of endogenous electrical fields^27^ and limitations of the EStim chamber we use in our laboratory,^24^ four different voltages (10, 50, 100, and 150 mV/mm) were selected. As was seen in previous studies on BM-MSCs and AT-MSCs, 10 mV/mm of EStim was found to have no significant effect on DPSC number and metabolic activity. In contrast, we observed that 100 and 150 mV/mm significantly reduced cell number and were cytotoxic. These findings indicate that DPSCs are more sensitive to EStim than the other MSCs tested in our previous experiments.^13,15^ Along these lines, Ramos et al. recently demonstrated that electrical impedance (resistance of cells to current flow) is decreased as mineral mass is increased in cell-mediated mineral constructs.^28^ Since DPSCs have been shown to have stronger mineralization capacity than BM-MSCs,^29^ perhaps, as differentiation occurs, electrical currents conduct more quickly and are sensed more intensely by DPSCs. In addition, another explanation for the observed effects could be differences between MSCs and DPSCs in their intercellular ion distribution and sensitivity to EStim-generated reactive oxygen species (ROS) in the medium (reviewed in Ref.^30^). As mentioned previously, higher voltages and long duration of EStim treatment can cause increased levels of ROS, which can decrease cell metabolic activity.^31^ The lower number of cells in the group treated with 100 mV/mm of EStim could explain the observed small increase in cell activity.

Several studies have demonstrated that EStim increases BM-MSC and AT-MSC osteogenic differentiation.^13,15,32,33^ Contrary to these findings, in this study we found that even though 50 mV/mm of EStim did not affect DPSC metabolic activity, it caused a decrease in differentiation parameters such as collagen and calcium deposition and ALP activity. Differences observed between our results and those of others could be related to the EStim regimen we used. It was shown that osteogenic differentiation positively correlates with EStim intensity,^33^ whereas exposure time can have the more complex correlations.^32^ Lower electrical resistance on polypyrrole (PPy) films was found to correlate with higher levels of mineralization in DPSCs.^23^

In our experimental setup DPSCs sense electrical stimuli through their surrounding culture medium, while in Cheng et al.'s^23^ setup the cells were in direct contact with the conductive surface of the PPy film. The makeup and resistance of a material from which cells sense an electrical stimuli can influence the cell's response to the stimuli.^34,35^ In our setup, the electrodes inserted in the culture medium can be a source of ROS, which can decrease cell metabolic activity when exposed for long periods.^31^ Results reported by Cheng et al.^23^ indicate that human DPSC EStim, applied once, in the early stages of differentiation, increases calcium deposition and expression of *BMP* genes. Our findings, in rat DPSCs, show that daily EStim treatment decreases odonto-/osteogenic differentiation. It is clear from the findings in both of these studies that the optimal EStim treatment regimen for DPSCs is yet to be determined. These studies contribute to a better understanding of how EStim affects DPSC behavior and thus provides a foundation for further studies aimed at using EStim to optimize DPSC odonto-/osteogenic function for clinical applications.

Another possible reason for the discrepancy observed between the results presented here and those reported by others could be due to inherent cell type/origin differences. Differences in proliferation rates, ALP activity, and responses to cytokine treatments between rat and human MSCs were previously reported.^36,37^ Studies have shown that MSCs derived from different sources display different characteristics. For example, they may differ in their ability to develop into distinct tissues. These characteristics are thought to be similar to and even determined by the microenvironment of their origin (reviewed in Ref.^38^). For example, stem cells from dental pulp, dental follicle, and periodontal ligament share several similarities in their DNA methylation patterns; however, they differ in their osteogenic potential.^39^ In previous studies we observed that despite EStim positively effecting osteogenic differentiation in both BM-MSCs and AT-MSCs, their osteogenic gene expression patterns differed markedly.^15^ This was also the case in the present study, where osteogenic marker gene expression patterns in EStim treated DPSCs differed from those of BM-MSCs and AT-MSCs treated with EStim. For example, *Osteopontin* gene expression was shown to be significantly affected by EStim in BM-MSCs and AT-MSCs,^13,15^ yet it was not affected by EStim in DPSCs. This might be due to differences in mechanisms and signaling, activated by EStim, in DPSC differentiation. Proteome of DPSCs could differ from other types of MSCs due to their different embryonic origin.^40^ This and other findings such as high levels of expression of *Osteocalcin* and *Col1a1* at day 14 and expression of the odontogenic gene markers *DSPP, BSP,* and *DMP1* at day 7 could also support the idea that cell populations derived from dental tissues appear to be more committed to odontogenic rather than osteogenic development (reviewed in Ref.^38^).

Even though we found that EStim treatment upregulates *RunX2*, *Osteocalcin*, *BSP*, and *DMP1* genes in DPSCs, at day 7 this was accompanied by structural changes and a decrease of collagen deposition, followed by a decrease of calcium deposition at day 14. This emphasizes that expression of these genes does not influence cell behavior alone and other genes and pathways, like those involved in extracellular matrix (ECM) formation, which might play a more important role in EStim induced DPSC differentiation.

Modifications of collagen crosslinking/network within ECM have been associated with decreased odontoblast differentiation and bone and dentin malformations (reviewed in Ref.^41^). It has been suggested that mineral constituents like phosphate ions are transported into bone and dentin collagen fibrils, thereby exerting great influence on molecular packing of collagen network and mineralization of tissues.^42^ Calcium accumulation, chelation, and mineralization of nodules of inorganic hydroxyapatite are main events during differentiation of MSCs into mineralized tissues (reviewed in Ref.^43^). Therefore, if these events are compromised, as observed in our EStim treated cells, proper osteo-/odontogenic differentiation does not occur.

Studies showing the response of DPSCs to EStim are still very scarce in the literature; therefore, this study, in a simple 2D model, will serve as an initial first step to gain a better understanding of how these cells respond to EStim. It is our hope that this initial knowledge will serve as a foundation for conducting further experiments in more complex 3D culture setups and in *in vivo* model systems. Further *in vitro* and *in vivo* studies need to be conducted with an eye toward translating these findings into better clinical treatments for patients.

The use of EStim in oral TE applications is gaining visibility among researchers and clinicians alike. However, further studies are needed to better define optimal EStim treatment regimens for DPSCs. From our findings we can conclude that, under the conditions of the present experiments, when applied at 50 mV/mm voltage for 7 and 14 days, EStim impairs DPSC osteo-/odontogenic differentiation. Despite odontogenic marker genes being upregulated by EStim treatment; deposition of extracellular components (collagen and calcium) and ALP activity were significantly reduced. Further experiments are needed to sort out the molecular mechanisms and signaling pathways involved in DPSC electrosensitivity. This knowledge could provide a better understanding of the underlying cellular mechanisms for the development of improved dental TE-based treatments.

## Supplementary Material

Supplemental data

## Abbreviations Used

ALP: alkaline phosphatase
AT-MSC: adipose-derived mesenchymal stem cell
BM-MSC: bone marrow derived mesenchymal stem cell
*BMP*: bone morphogenic protein
*BSP*: bone sialoprotein
*Col1a1*: collagen type 1 alpha 1
DC: direct current
DMEM: Dulbecco's modified Eagle's medium
*DMP1*: dentin matrix protein-1
DPSC: dental pulp stem cell
*DSPP*: dentin sialophosphoprotein
ECM: extracellular matrix
EStim: electrical stimulation
MSC: mesenchymal stem cell
PBS: phosphate-buffered saline
PCR: polymerase chain reaction
PFA: paraformaldehyde
PPy: polypyrrole
ROS: reactive oxygen species
*RPLP*: ribosomal protein P1
RQ: relative quantification
RT-qPCR: reverse transcriptase quantitative polimerase chain reaction
*RunX2*: Runt-related transcription factor 2
TE: tissue engineering
*YWHAZ*: tyrosine 3-monooxygenase/tryptophan 5-monooxygenase activation protein zeta
