# Supplementary material for: Electrical Stimulation Decreases Dental Pulp Stem Cell Osteo-/Odontogenic Differentiation
Source: Biores Open Access. 2020 May 27;9(1):162–73. doi: 10.1089/biores.2020.0002 (PMC7337168; doi:10.1089/biores.2020.0002)
Supplement: Supplemental data [file Suppl_Data.pdf]

Supplementary Data

Supplementary Methods

Cell isolation and culture

The mandibles of donor rats were dissected, cleaned, and disinfected with 0.2% chlorhexidine solution (Dynexidin Forte; Chemische Fabrik Kreussler & Co. GmbH, Wiesbaden, Germany). Dental pulp tissue was extracted using endodontic files (Nos.: 25–45, Gebr. Brasseler GmbH & CO. KG, Lemgo, Germany), cut, and incubated in 3% collagenase solution (Bio-Chrom GmbH, Berlin, Germany) in Dulbecco's modified Eagle's medium (DMEM) + GlutaMAX +1 g/L D-glucose (Gibco, Gaithersburg, MD, USA) for 45 min at 37°C. The cell suspension was precipitated at 300 × *g* for 10 min, the resulting cell pellet was dissolved in cell growth medium consisting of DMEM +10% fetal bovine serum and 1% penicillin/streptomycin (10,000 U/mL), all obtained from Gibco (Gaithersburg, MD, USA), and cultured in a humidified incubator at 37°C, 5% CO_2. When 80% confluence was reached, cells were expanded over three to six passages to achieve appropriate cell number. Medium change was performed twice weekly.

Characterization of isolated cells

Phenotype characterization. Isolated dental pulp stem cells (DPSCs) (2×10^5 cells), at passage 3, were resuspended in 100 μL of growth medium and incubated with primary antibodies, anti-rat CD90 FITC, anti-rat CD45 APC, both purchased from eBioscience, Inc. (San Diego, USA), and anti-CD34 FITC from Bioss Antibodies, Massachusetts, USA, for 20 min at 4°C in the dark. Cells were washed with phosphate-buffered saline (PBS), precipitated for 5 min at 300 × *g*, and resuspended in 350 μL of PBS for analysis. 7AAD (BD Biosciences) staining was used to visualize and confirm cell viability. Flow cytometry analysis was performed using FACSCalibur (Becton-Dickinson Biosciences, Heidelberg, Germany). Events were gated (light scatter) to exclude dead cells and debris. Fluorescence data were acquired from at least 10,000 events in the viable cell gate and analyzed using Cell Expert software (Becton-Dickinson Biosciences).

Multilineage differentiation. For osteo-/odontogenic differentiation, DPSCs were seeded at a density of 1.25×10^4 cells/cm^2 in a six-well plate (TPP, Trasadingen, Switzerland) in growth medium, and from the second day on, the cell growth medium was supplemented with 10^{-7} M dexamethasone, 10 mM β-glycerophosphate, and 0.05 mM ascorbic acid-2-phosphate, all obtained from Sigma-Aldrich (Heidelberg, Germany). Cells were cultured at 5% CO_2 and 37°C for 21 days during which time the medium was changed every 3 days. Alizarin Red staining was used as an indicator of extracellular matrix calcification, confirming cell osteogenic differentiation. DPSCs were washed twice with PBS and fixed with 4% paraformaldehyde (PFA) (Merck KGaA, Darmstadt, Germany) solution in PBS for 15 min. Alizarin Red S (Sigma-Aldrich, München, Germany) solution (2% in PBS) was added to the fixed cells; cells were incubated at room temperature for 30 min and rinsed with deionized water repeatedly. Images of stained cells were captured with a light microscope (CKX41, cellSens Imaging system; Olympus, Tokyo, Japan) at a magnification of 10×.

For chondrogenic differentiation, 2×10^5 cells, suspended in 500 μL growth medium, were seeded into 96-well plates (Sarstedt, Nümbrecht, Germany), precipitated at 2000 rpm for 5 min, to form a pellet, and incubated at 5% CO_2 and 37°C. After 24 h, growth medium was supplemented with 100 mM dexamethasone, 200 μM ascorbate-2 phosphate, 10 ng/mL insulin-transferrin-sodium selenite (ITS-3) (all from Sigma LifeSciences, Darmstadt, Germany), and 10 ng/mL TGF-β3 (R&D Systems, Minnesota, USA), and cell pellets were cultured for 21 days. Induction medium was freshly prepared and changed every 2 days. At day 21, cell pellets were fixed in 4% PFA solution for 1 h, incubated in increasing concentrations of sucrose solutions (10%, 20%, and 30%), embedded in Tissue-Tek O.C.T compound (Sakura Finetek Europe BV, Netherland) for cryopreservation, and stored at −80°C until they were sectioned. The frozen pellets were sectioned using CryoStar Nx50 Cryostat (Thermo Fisher Scientific, MA, USA), at −19°C, at a thickness of 10 μm, and mounted on glass microscope slides. Pellet sections were stained with dimethylmethylene blue solution (Sigma-Aldrich, München, Germany), for 3 min, dehydrated with isopropanol (80%, 90%, and 100%), cleared in xylol (all purchased from Carl Roth GmbH, Karlsruhe, Germany), and mounted (Roti®-Histokitt II, Carl Roth GmbH). Stained sections were imaged and

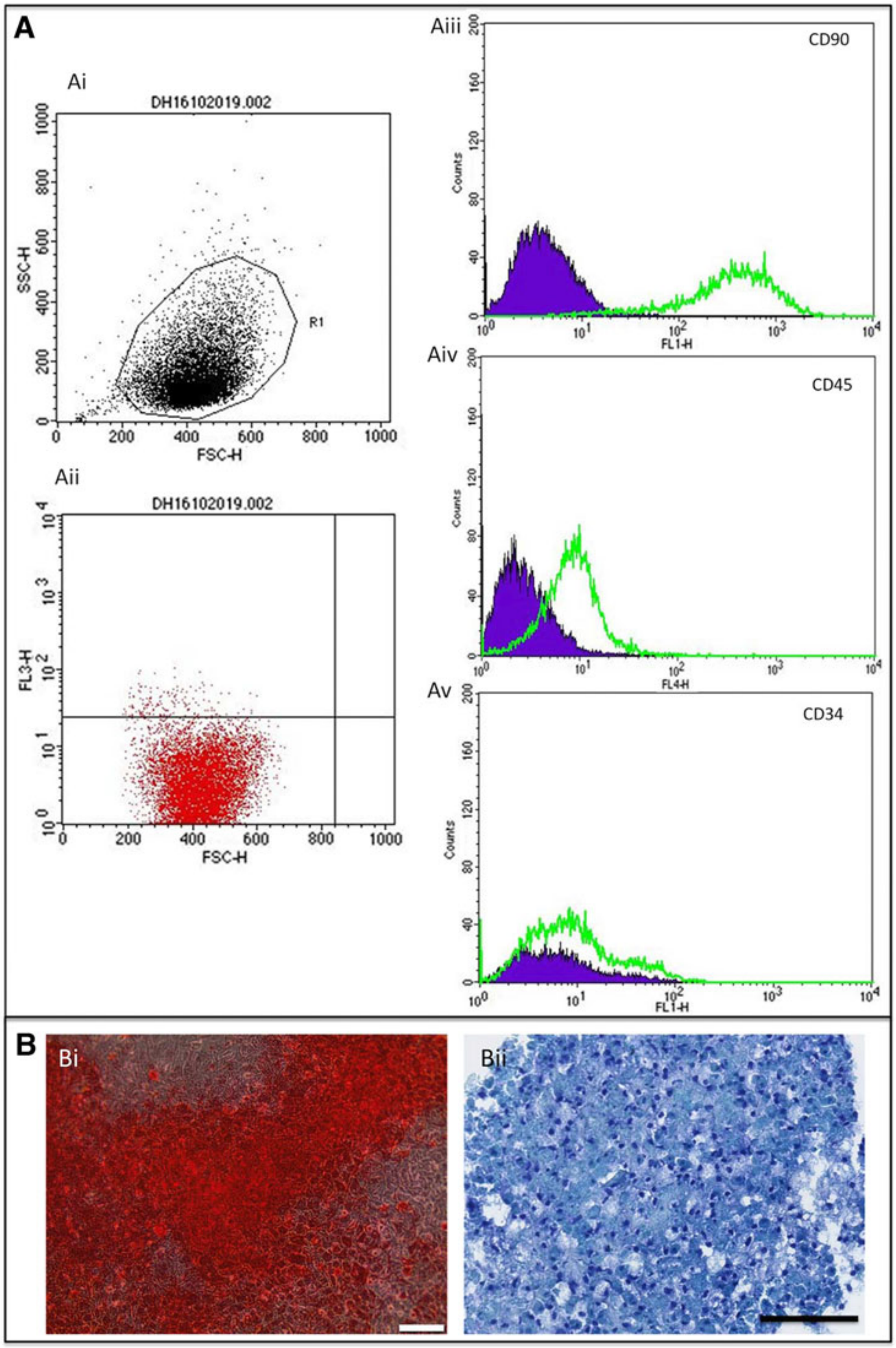

SUPPLEMENTARY FIG. S1. Isolation and characterization of rat DPSCs. **(A)** Characterization of DPSC surface marker profiles using flow cytometry analysis. **(Ai)** Cell morphology was assessed by flow cytometry according to forward/side scatter characteristics; **(Aii)** Cell viability (98.2%) was assessed by means of 7AAD staining. **(Aiii–Av)** Histograms represent expression of cell surface markers CD90, CD45, and CD34, respectively. Violet peaks indicate unstained DPSCs; green lines represent DPSCs stained with the indicated antibodies. **(B)** Multilineage differential potential of isolated DPSCs. **(Bi)** DPSCs stained with Alizarin Red showing calcium deposition after 21 days culture in osteo-/odontogenic conditions (10 × magnification. Scale bar = 200 μm). **(Bii)** DMMB staining of glycosaminoglycans in DPSC-pellet after 21 days culture in chondrogenic medium (10 × magnification. Scale bar = 100 μm). DMMB, dimethylmethylene blue; DPSC, dental pulp stem cell.

Supplementary Table S1. Groups Used to Determine the Effects of 50 mV/mm Electrical Stimulation on Osteo-/Odontogenic Differentiation of Dental Pulp Stem Cell

Groups	*EStim treatment*	*Time points*	*Measurements*
EStim	50 mV/mm 1 h/day	Days 0, 7, and 14	Collagen and calcium deposition<br>ALP activity<br>Gene expression
Control	No EStim	Days 0, 7, and 14	Collagen and calcium deposition<br>ALP activity<br>Gene expression

ALP, alkaline phosphatase; EStim, electrical stimulation.

analyzed using light microscopy (Ti-E, Nikon GmbH, Germany) and image analysis software (NIS-Elements 4.4; Nikon GmbH, Germany).

Electrical stimulation of DPSCs

Cell number. Cells were lysed (400 mM potassium phosphate buffer, 2% Triton X-100, 10 mM EDTA, pH7.0, all purchased from SIGMA-Aldrich) and sonicated (45 KHz, 10 min, +4°C). The samples were stained with PicoGreen reagent, excited at 480 nm, and the intensity of fluorescence was measured at 520 nm using a spectrofluorometer (Infinite 200PRO; Tecan, München, Germany). A calibration curve, developed from cell lysates with known number of cells, was used to calculate cell number for each well.

Cell metabolic activity. AlamarBlue reagent was added to cells (10% final concentration) and incubated for 4 h at 37°C in the dark. Absorbance was measured (570 and 600 nm) in triplicates for each well using an Infinite 200 PRO reader. Percentage of reduction was calculated, and values were normalized to the number of cells for each well. The mean value for three wells was calculated for each group and used for statistical analysis.

Assessment of DPSC osteo-/odontogenic differentiation

Collagen formation. Cells were washed twice with PBS, fixed with methanol (Sigma-Aldrich) overnight at room temperature, and stained for 1 h with Picrosirius Red solution (0.1% in saturated aqueous solution of picric acid; Sigma Aldrich). Acetic acid solution (0.1%) was used for repeated rinse, and cells were imaged using fluorescence microscope (TexRed filter) (Eclipse Ti inverted; Nikon Instruments, Tokyo, Japan).

Alkaline phosphatase activity. For detection of alkaline phosphatase (ALP) activity, p-Nitrophenyl phosphate (pNPP) chromogenic substrate absorbance was measured at 405 nm with an Infinite 200 PRO plate reader (Tecan, München, Germany). The calibration curve of *p*-nitrophenol standards was used to calculate the amount of ALP for each well. Fold change in ALP activity was obtained after normalization to absorbance values obtained in the same way from cells collected 1 day after seeding (day 0).

Osteo-/odontogenic marker gene expression analysis. Total RNA was isolated from cells using Aurum Total RNA Mini Kit (Bio-Rad, Germany). Quality and quantity of RNA were evaluated by gel electrophoresis and Infinite 200 PRO NanoQuant device, respectively. cDNA synthesis was conducted using a qScript cDNA Synthesis Kit (QuantaBio, Massachusetts, USA) according to the manufacturer's recommendations. Quantitative real-time polymerase chain reaction (PCR) was performed using a cDNA equivalent of 5 ng RNA and SsoAdvanced Universal SYBR Green Supermix (Bio-Rad, Germany). All

Supplementary Table S2. qRT-Polymerase Chain Reaction Primer Sequences

Gene	*Forward primer*	*Reverse primer*
BSP	AGAAATTGCAAAATGAAGACTGCT	GCGTGGCCGTACTTAAAGA
Col1a1	GTACATCAGCCCAAACCCCA	TCGCTTCCATACTCGAACTGG
DMP1	CGCCCATGGCAAATAGTGAC	CTCCTTATCGGCGTCCATCC
DSPP	ATCTGCCGACGTACCCTTTC	ATCGTCGTTAGTGGCGTTGT
Osteopontin	GATGAACAGTATCCCGATGCC	TCCAGCTGACTTGACTCATGG
Rplp1	GCATCTACTCCGCCCTCATC	GCATCTACTCCGCCCTCATC
RunX2[a]	[a]	[a]
Ywhaz	GATGAAGCCATTGCTGAACTTG	GTCTCCTTGGGTATCCGATGTC

BSP, bone sialoprotein; *Col1a1*, collagen type 1 alpha 1; *DMP1*, dentin matrix protein 1; *DSPP*, dentin sialophosphoprotein; *Rplp1*, Ribosomal protein P1; *RunX2*, Runt-related transcription factor 2; *Ywhaz*, tyrosine 3-monooxygenase/tryptophan 5-monooxygenase activation protein zeta.

[a]*RunX2* purchased from Qiagen, Germany (Catalog No. 330523).

samples were amplified in duplicates with a CFX96 Touch Real-Time PCR Detection System (Bio-Rad) using rat gene specific primers (Supplementary Table S2). Ribosomal Protein P1 (*Rplp1*) and Tyrosine 3- monooxygenase/tryptophan 5-monooxygenase Activation Protein Zeta (*Ywhaz*) were used as reference genes. A melting curve analysis was applied to ensure specificity of the PCR products. Amplification products were also analyzed by gel electrophoresis. Relative quantification (RQ) of messenger RNA levels of the target genes was analyzed using the comparative CT (threshold cycle values) method ($2^{-\Delta\Delta Ct}$).[S1] The results are presented as RQ, which is the fold change in expression compared to the housekeeping genes and expression of specific gene markers at day 0. Three samples were analyzed for each group, and the mean values and standard deviation were calculated for further analysis.

Supplementary Reference

S1. Livak KJ, Schmittgen TD. Analysis of relative gene expression data using real-time quantitative PCR and the 2-ΔΔCT method. Methods. 2001;25:402–408.
